# A novel method of blind bedside placement of postpyloric tubes

**DOI:** 10.1186/s13054-018-1986-0

**Published:** 2018-03-09

**Authors:** Jia-Kui Sun, Xiang Wang, Shou-Tao Yuan

**Affiliations:** Department of Intensive Care Unit, Nanjing First Hospital, Nanjing Medical University, 68 Changle Road, Nanjing, Jiangsu Province, 210006 China

## ᅟ

We read with great interest the recent report on blind bedside postpyloric placement by Lv et al. [1]. Their methods were proven to be safe and effective in intensive care units. Although our placing procedure is similar to that reported by the authors, the choice of a postpyloric tube and the patient’s position requires further improvement.

**Fig. 1:**
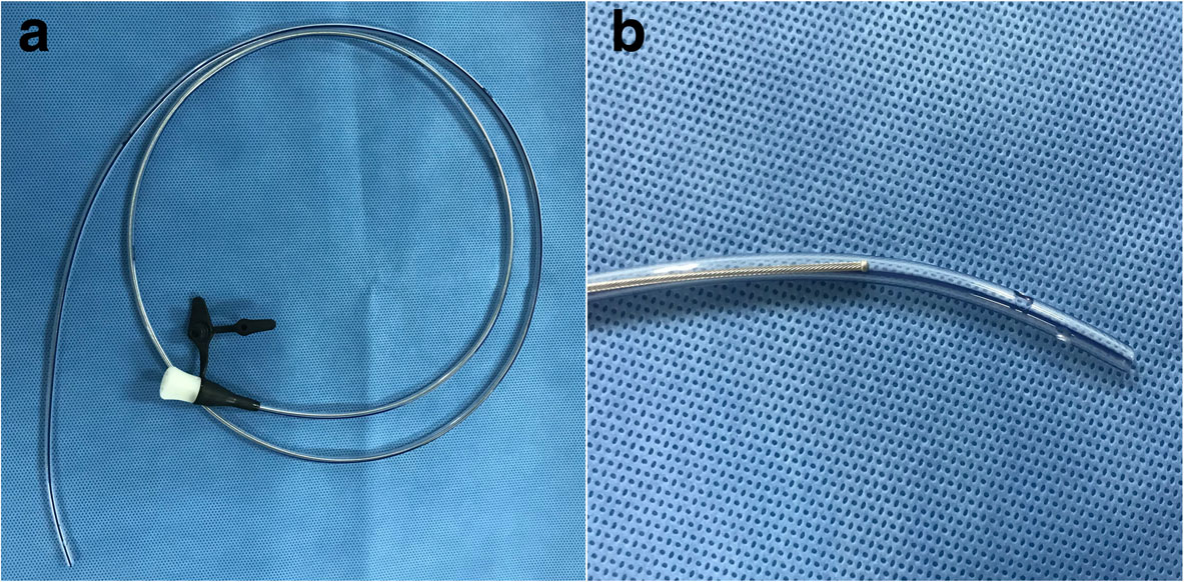
The 130-cm long transpyloric tube with a guidewire (CH10–130, inner diameter 2.0–2.1 mm, Flocare, Nutricia Ltd, Wuxi, China) used in our center (**a**). This Flocare tube has two side holes near its tip (**b**)

In our center, we use a 130-cm long transpyloric tube with a guide wire (CH10–130, inner diameter 2.0–2.1 mm, Flocare, Nutricia Ltd, Wuxi, China; Fig. 1a) rather than the spiral feeding tube used by Lv et al. The Flocare tube that we used has several advantages compared with the spiral tube. First, the Flocare tube is inexpensive, approximately $22, in China, whereas the spiral tube costs approximately $71. Expense is extremely important in developing countries and at one-third less compared with the latter, our technique is easier to implement in hospitals and areas with limited resources. Second, the Flocare tube that we used has two side holes near its tip (Fig. 1b); therefore, it is less likely to be blocked compared with the spiral tube. Third, the guide wire is shorter in length compared with the Flocare tube; thus, the rigid tip would not damage the digestive tract during the placing procedure.

The patient’s position before placing the postpyloric tube also requires further improvement. In our procedure, the patient is placed in a right decubitus position at 30–45° after gastric placement is accomplished, followed by a postpyloric placement. According to our experience, in this position the tip of the Flocare tube falls to the pylorus ostium by gravity, which may increase the placement success rate.

**Fig. 2:**
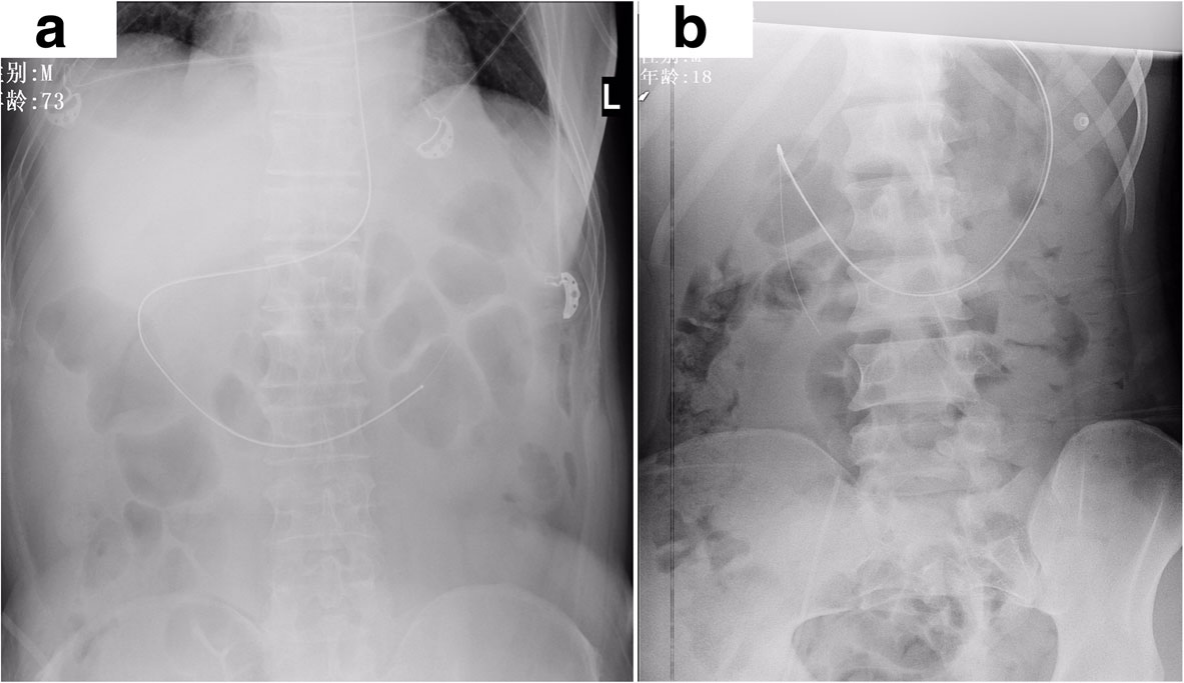
Abdominal plain radiographs showing the tip of the Flocare tube positioned at the horizontal part (**a**) and descending part (**b**) of the duodenum. The former demonstrates the classic C-shaped duodenal configuration diagnostic of postpyloric placement

The results of our data (unpublished) analysis confirmed our improved methods (Fig. 2). From December 2016 to December 2017, 44 patients underwent postpyloric tube placement using our novel techniques. In total, 38 cases (86.4%) were successful, and 33 cases (75.0%) were successful at the first attempt. The success rate and first-time success rate of our placement techniques were better than those described in the study by Lv et al. The median time of our procedure was 13 (8.5–16) minutes, and the median insertion length was 100 (93.5–110) cm. These values are similar to the results of Lv et al. and other previous reports [1–3].

Considering the less expensive tube and better first-time success rate, our novel blind bedside postpyloric placement may be easier to implement worldwide, and we look forward to collaborating with the authors and other colleagues.
